# Supplementary material for: A novel incision design for Vibrant SoundBridge®︎ implantation prior to auricular reconstruction for microtia
Source: PLoS One. 2025 Jul 29;20(7):e0327240. doi: 10.1371/journal.pone.0327240 (PMC12306728; doi:10.1371/journal.pone.0327240)
Supplement: Attachment — Submitted filename: Response to Reviewers.docx [file pone.0327240.s002.docx]

**Reviewer #1:**

Thank you very much for your thoughtful comments and questions. We have addressed each of them below.

***At line 139
"A subcutaneous pocket was created through standard dissection"
You did not mention which incision was used to access the pocket.***

→We have added the following text to the main body of the manuscript.

“A W-shaped skin incision was made.”

***In table 1 last column
It is other congenital anomalies and not other complications***

→We have revised the wording from “other complications” to “other congenital anomalies.”

***In figure 5a the scar of insertion of VSB is not visible***

→We have added a photograph that clearly shows the scar from the VSB implantation, now presented as Fig. 5b.

***Please add to the limitation of the study small number of cases***

→We have added the following text to the main body of the manuscript.

“Third, although no flap necrosis or device damage was observed in this study, the small sample size precluded statistical analysis, and further case accumulation is warranted for future evaluation.”

**Reviewer #2:**

***Authors should just enrich the "discussion" section by briefly mention two concepts:***

1) ***the role of technology for the assessment of prognosis in contemporary Plastic Surgery (for every field of this Discipline, from wounds to microsurgery)***

→ Thank you for suggesting those references. We have carefully reviewed the papers you recommended, which describe methods for ulcer assessment using devices and scoring systems. These are indeed highly interesting contributions to the field of wound management. While enabling prognostic evaluation of wounds is undoubtedly an important challenge in wound care, it lies beyond the primary scope of our current manuscript. Therefore, we have decided not to include additional discussion on this topic.

2) ***the potential future expansion of this kind of method for post oncologic
   auricular reconstruction.***

→ Thank you for your valuable comments. We have added the following text to the manuscript to address the applicability of our method to acquired auricular defects, including those resulting from tumor resection or trauma. We have also cited the references you kindly suggested.

“Various reconstructive techniques have been described for patients with acquired auricular defects following tumor resection or trauma. We believe our method is unsuitable for these patients. In these cases, VSB implantation is generally not performed prior to auricular reconstruction. Instead, it is technically simpler and preferable to implant the VSB either after auricular reconstruction or concurrently with auricular elevation.”
